# Tungsten carbide cobalt nanoparticles exert hypoxia-like effects on the gene expression level in human keratinocytes

**DOI:** 10.1186/1471-2164-11-65

**Published:** 2010-01-27

**Authors:** Wibke Busch, Dana Kühnel, Kristin Schirmer, Stefan Scholz

**Affiliations:** 1 UFZ - Helmholtz-Centre for Environmental Research Leipzig, Department of Bioanalytical Ecotoxicology, Permoserstr. 15, 04318 Leipzig, Germany; 2 Eawag, Swiss Federal Institute of Aquatic Science and Technology, 8600 Dübendorf, Switzerland and ETH Zürich, Institute of Biogeochemistry and Pollutant Dynamics, 8092 Zürich, Switzerland

## Abstract

**Background:**

Tungsten carbide (WC) and tungsten carbide cobalt (WC-Co) nanoparticles are of occupational health relevance because of the increasing usage in hard metal industries. Earlier studies showed an enhanced toxic potential for WC-Co compared to WC or cobalt ions alone. Therefore, we investigated the impact of these particles, compared to cobalt ions applied as CoCl_2_, on the global gene expression level in human keratinocytes (HaCaT) *in vitro*.

**Results:**

WC nanoparticles exerted very little effects on the transcriptomic level after 3 hours and 3 days of exposure. In contrast, WC-Co nanoparticles caused significant transcriptional changes that were similar to those provoked by CoCl_2_. However, CoCl_2 _exerted even more pronounced changes in the transcription patterns. Gene set enrichment analyses revealed that the differentially expressed genes were related to hypoxia response, carbohydrate metabolism, endocrine pathways, and targets of several transcription factors. The role of the transcription factor HIF1 (hypoxia inducible factor 1) is particularly highlighted and aspects of downstream events as well as the role of other transcription factors related to cobalt toxicity are considered.

**Conclusions:**

This study provides extensive data useful for the understanding of nanoparticle and cobalt toxicity. It shows that WC nanoparticles caused low transcriptional responses while WC-Co nanoparticles are able to exert responses similar to that of free cobalt ions, particularly the induction of hypoxia-like effects via interactions with HIF1α in human keratinocytes. However, the enhanced toxicity of WC-Co particles compared to CoCl_2 _could not be explained by differences in gene transcription.

## Background

Engineered nanomaterials are used in large amounts in several industries and an increasing demand, including new types of particles, is anticipated in the future [1]. Their physico-chemical properties, i.e. the small size and the high surface to volume ratio are one of the most interesting characteristics, which is useful for many applications in medicine, chemistry, material sciences and physics. However, these physico-chemical characteristics may be associated with undesired health effects not known for, or different from, the bulk materials. Hence, the field of nanotoxicology is emerging to assess possible hazards of nanomaterials. Several reviews have summarised the potential cellular mechanisms of nanoparticles toxicity such as increase in the production of reactive oxygen species (ROS) and induction of inflammatory responses [2-4]. The cellular responses appear to be dependent on the physical and chemical properties of the particles, such as particle size, dissolution behaviour, surface reactivity and binding ability [2,5,6].

So far, the majority of *in vivo *and *in vitro *studies in nanotoxicology have focussed on endpoints such as vitality, production of reactive oxygen species, immunological parameters or cell death. However, the elucidation of the mode of action and identification of subacute effects with potential implications for chronic toxicity are difficult to obtain from these studies. Therefore, modern toxicogenomic approaches established already in pharmacology and toxicology [7-10] could be used to unravel the toxicodynamics of nanomaterials. First studies on the effects of nano- or ultrafine particles on global gene expression patterns revealed compound-specific but no general responses due to the exposure to particles [11-14]. Hence, the chemical composition of the particles seems to play a major role for transcriptional responses. Griffitt and colleagues [11] showed that metal ions (silver and copper) caused similar expression patterns as nanoparticles of the same materials in zebrafish, but the numbers of affected genes were always higher after exposure to the particles. In an *in vitro *study by Waters et al. [12] it was found that changes in cell viability provoked by silica exhibited a higher correlation with particle surface area than either particle mass or number in macrophages. The majority of biological processes represented by the differentially expressed genes were nearly identical, irrespective of particle diameter.

A toxicogenomic approach has been used in this study to analyse the mode of action of tungsten carbide (WC) and tungsten carbide cobalt (WC-Co) nanoparticles. These nanoparticles are intended to be increasingly used in hard metal industries for the production of wear resistant and hard tools. The major advantage of using WC and WC-Co nano-scaled instead of micro-scaled particles is the increased hardness of resulting composite materials and therefore a prolonged wear lifespan of tools and other products [15]. Cobalt serves as binding agent improving the sintering of hard metals from WC nanoparticles. Therefore, the use of WC-Co particles is favoured in hard metal industries. Potential health implications may be of concern for workers involved in the manufacturing process. Previous studies using μm-sized particles have indicated a lack of toxicity for WC particles but a hazardous potential for cobalt metal particles *in vivo *and *in vitro *[16-18]. A mixture of these μm-scaled powders (WC-Co) exhibited an enhanced toxicity if compared to cobalt metal powder alone [16-20]. Our previous research also showed toxicity enhancing effects for nano-sized WC-Co compared to WC or CoCl_2 _[21,22]. The increased toxicity was proposed to result from specific interactions of WC and cobalt. Since the International Agency for Research on Cancer (IARC) has classified cobalt as 'possibly carcinogenic' and tungsten carbide cobalt as 'probably carcinogenic' to humans [23] research on the elucidation of the mode of action of nano-sized particles of these materials is of high relevance for occupational health. Ionic cobalt (Co^2+^) is known to exert hypoxia like responses via stabilising the α subunit of the hypoxia inducible transcription factor (HIF1) [24-26]. Ubiquitously expressed HIF1α is degraded via oxygen-dependent prolyl-4-hydroxylation under normoxic conditions [27]. These degradation processes are blocked by cobalt binding or oxygen deficiency (hypoxia) which results in enriched HIF1α levels in the cells. HIF1 is a transcription factor that mediates response to hypoxia by regulating the transcription of genes encoding proteins that play key roles in angiogenesis, glucose and energy metabolism, cell survival and proliferation, iron metabolism, and vascular functions [28,29]. Comparative gene expression studies showed HIF1-mediated responses to be similar for hypoxia and CoCl_2 _exposure [8,30].

Whether metallic cobalt in nanoparticles, particularly in combination with tungsten, provokes specific toxic effects deviating from or exceeding those observed for dissolved ionic cobalt is not finally clarified yet. Lison and co-workers [31] described the formation of reactive oxygen species (ROS) after a rapid dissolution of cobalt ions out of WC-Co micrometer sized particles in a cell free system, a phenomenon that could not be found with metal cobalt particles or a combination of WC particles with CoCl_2_. Furthermore, another study that evaluated the role of ROS in the interactive toxicity of carbide-cobalt mixtures found no evidence that production of ROS contributed to the toxicity of WC-Co in macrophages [32]. Lombaert and co-workers [33] investigated gene expression in macrophages exposed to micrometer sized WC-Co particles (a mixture of cobalt metal with a median particle size (d_50_) of 4 μm and WC particles d_50 _< 1 μm). They identified differential expression of genes involved in apoptosis regulation, stress response, glucose metabolism, cell signalling, immune response and other pathways. The effects were discussed to be at least partially provoked by dissolved cobalt ions.

In a previous study [21] we have investigated the impact of WC and WC-Co nanoparticles on the vitality of various mammalian cells (lung, skin, colon and oligodendrocyte cell lines; primary neural cell and astroglial cultures). Significant cytotoxic effects were observed for nano-sized WC-Co (33 μg/ml). About 15% of WC and 76% of Co were found to be dissolved after 1 week of storage of the stock solution. Interestingly, WC-Co particles (consisting of 30 μg/ml WC and 3 μg/ml cobalt) showed a higher toxicity than equivalent concentrations of CoCl_2 _(3 μg/ml) indicating that leaching of cobalt alone may not explain the toxic effects. It was also demonstrated that the tungsten based nanoparticles could enter various cell types [21,22]. Based on this study we selected the human skin cell line HaCaT to investigate the effects of WC and WC-Co nanoparticles and cobalt ions on gene expression patterns. Our major goal was to elucidate (1) whether the observed effects indicate specific mode of actions of WC-Co nanoparticles and/or (2) whether the effects can primarily be explained by dissolved Co.

## Results

We compared the mode of action of WC and WC-Co nanoparticles and dissolved CoCl_2 _in HaCaT cells by recording changes in transcription profiles by microarray analysis. HaCaT cells were exposed to the lowest concentration of WC-Co causing a reduction of cell vitality (33 μg/ml; [21]) and corresponding concentrations of WC and CoCl_2_. RNA isolated after 3 h and 3 d of exposure from 5 independent biological replicates per treatment was analysed using a commercial human whole genome microarray. Various analyses routines were performed to identify differentially expressed genes, treatment clusters and affected biological pathways.

### Identification of differentially expressed genes

SAM analysis of normalised microarray fluorescence intensities for all treatments revealed 1956 significantly differentially expressed genes with about 1146 showing an induction or repression of more than 2fold. The highest number of genes with a significantly altered expression above 2fold was observed after 3 d of exposure (Table 1). Among the different treatments, exposure to CoCl_2 _provoked the strongest changes in gene expression (373 and 826 genes for 3 h and 3 d of exposure, respectively) followed by WC-Co (37 and 248, respectively) and WC nanoparticles (28 and 49 respectively). Comparison of the genes affected by the different treatments revealed a considerable overlap of transcription profiles. The highest commonalities were observed between the gene expression patterns of CoCl_2 _and WC-Co after 3 d of exposure (184 genes differentially expressed in both treatments), followed by the exposure to CoCl_2 _at 3 h and 3 d (134 genes) and WC/WC-Co at 3 d (31 genes). A list of the genes with the strongest differential expression (>5fold) can be found in Table 2, the complete set of genes is available in the Additional file 1.

**Table 1:** Number of significant repressed or induced genes > 2fold per treatment*.

Treatment	up	down	total	Treatment	Overlapping genes between treatments				
WC3h	26	2	28	WC3h	WC3h				
							
WCCo3h	13	24	37	WCCo3h	8	WCCo3h			
						
CoCl3h	242	131	373	CoCl3h	8	17	CoCl3h		
					
WC3d	18	31	49	WC3d	3	2	11	WC3d	
				
WCCo3d	141	107	248	WCCo3d	2	8	29	31	WCCo3d
				
CoCl3d	541	285	826	CoCl3d	8	15	134	19	184

* identified with SAM; the pair wise comparison indicates genes that were differentially expressed in both of the considered treatments

**Table 2:** Genes with most prominent changes in expression levels^#^.

WC 3 h	WCCo 3 h	CoCl2 3 h	WC 3 d	WCCo 3 d	CoCl2 3 d	Gene Name	Accession	Description
	**up**	**up**	**up**	**up**	**up**	PPFIA4	NM_015053	protein tyrosine phosphatase, receptor type, f polypeptide (PTPRF), interacting protein (liprin), alpha 4 (PPFIA4), mRNA [NM_015053]

		**up**	**up**	**up**	**up**	BNIP3	NM_004052	BCL2/adenovirus E1B 19 kDa interacting protein 3 (BNIP3), nuclear gene encoding mitochondrial protein, mRNA [NM_004052]

		**up**	**up**	**up**	**up**	PDK1	NM_002610	pyruvate dehydrogenase kinase, isozyme 1 (PDK1), nuclear gene encoding mitochondrial protein, mRNA [NM_002610]

			**up**	**up**	**up**	CA9	NM_001216	carbonic anhydrase IX (CA9), mRNA [NM_001216]

			**up**	**up**	**up**	EGLN3	NM_022073	egl nine homolog 3 (C. elegans) (EGLN3), mRNA [NM_022073]

			**up**	**up**	**up**	LOXL2	NM_002318	lysyl oxidase-like 2 (LOXL2), mRNA [NM_002318]

			**up**	**up**	**up**	THC2369600	THC2369600	ALU6_HUMAN (P39193) Alu subfamily SP sequence contamination warning entry, partial (12%) [THC2369600]

				**up**	**up**	PLOD2	NM_182943	procollagen-lysine, 2-oxoglutarate 5-dioxygenase 2 (PLOD2), transcript variant 1, mRNA [NM_182943]

				**up**	**up**	PPP1R3C	NM_005398	protein phosphatase 1, regulatory (inhibitor) subunit 3C (PPP1R3C), mRNA [NM_005398]

				**up**	**up**	CST6	NM_001323	cystatin E/M (CST6), mRNA [NM_001323]

				**up**	**up**	FN1	NM_212482	fibronectin 1 (FN1), transcript variant 1, mRNA [NM_212482]

				**up**	**up**	THC2316753	THC2316753	Q91TG6 (Q91TG6) T130, partial (7%) [THC2316753]

				**up**	**up**	VLDLR	NM_003383	very low density lipoprotein receptor (VLDLR), transcript variant 1, mRNA [NM_003383]

				**up**	**up**	AML1a	ENST00000358356	mRNA for AML1a protein, complete cds. [D43967]

				**up**	**up**	HK2	NM_000189	hexokinase 2 (HK2), mRNA [NM_000189]

				**up**	**up**	PTGS2	NM_000963	prostaglandin-endoperoxide synthase 2 (prostaglandin G/H synthase and cyclooxygenase) (PTGS2), mRNA [NM_000963]

				**up**	**up**	SLC2A14	BC060766	solute carrier family 2 (facilitated glucose transporter), member 14, mRNA (cDNA clone MGC:71510 IMAGE:5297510), complete cds. [BC060766]

				**up**	**up**	GPI	NM_000175	glucose phosphate isomerase (GPI), mRNA [NM_000175]

				**up**	**up**	ENO2	NM_001975	enolase 2 (gamma, neuronal) (ENO2), mRNA [NM_001975]

				**up**	**up**	CDH2	NM_001792	cadherin 2, type 1, N-cadherin (neuronal) (CDH2), mRNA [NM_001792]

				**up**	**up**	AK021874	ENST00000366930	cDNA FLJ11812 fis, clone HEMBA1006364. [AK021874]

	**up**	**up**		**up**	**up**	ASB2	NM_016150	ankyrin repeat and SOCS box-containing 2 (ASB2), mRNA [NM_016150]

	**up**	**up**		**up**	**up**	ANGPTL4	NM_139314	angiopoietin-like 4 (ANGPTL4), transcript variant 1, mRNA [NM_139314]

		**up**		**up**	**up**	LOC653068	XM_925841	PREDICTED: similar to TBP-associated factor 9L (LOC653068), mRNA [XM_925841]

		**up**			**up**	TCF19	BC033086	transcription factor 19 (SC1), mRNA (cDNA clone MGC:45652 IMAGE:3160434), complete cds. [BC033086]

		**up**			**up**	AIPL1	NM_014336	aryl hydrocarbon receptor interacting protein-like 1 (AIPL1), transcript variant 1, mRNA [NM_014336]

		**up**			**up**	LOC200726	XM_117266	PREDICTED: hypothetical LOC200726 (LOC200726), mRNA [XM_117266]

		**up**			**up**	LUZPP1	AJ312775	mRNA for leucine zipper protein 3 (LUZP3 gene). [AJ312775]

		**up**				C9orf65	NM_138818	chromosome 9 open reading frame 65 (C9orf65), mRNA [NM_138818]

		**up**				THC2411387	THC2411387	ALU8_HUMAN (P39195) Alu subfamily SX sequence contamination warning entry, partial (9%) [THC2411387]

		**up**				SOX6	NM_033326	SRY (sex determining region Y)-box 6 (SOX6), transcript variant 2, mRNA [NM_033326]

		**up**				SULT2A1	NM_003167	sulfotransferase family, cytosolic, 2A, dehydroepiandrosterone (DHEA)-preferring, member 1 (SULT2A1), mRNA [NM_003167]

		**up**				C1orf67	BC042869	cDNA clone IMAGE:5270407. [BC042869]

		**up**				CRB1	NM_201253	crumbs homolog 1 (Drosophila) (CRB1), mRNA [NM_201253]

	**up**	**up**				EDN2	NM_001956	endothelin 2 (EDN2), mRNA [NM_001956]

					**up**	CBLN4	NM_080617	cerebellin 4 precursor (CBLN4), mRNA [NM_080617]

					**up**	G65686	ENST00000332107	A117 Human STS cDNA, sequence tagged site. [G65686]

					**up**	XAGE2	NM_130777	X antigen family, member 2 (XAGE2), mRNA [NM_130777]

					**up**	PELO	AF118075	PRO1770 mRNA, complete cds. [AF118075]

					**up**	SAA3P	AY209188	truncated serum amyloid A3 precursor (SAA3) mRNA, complete cds. [AY209188]

					**up**	ITIH5	NM_030569	inter-alpha (globulin) inhibitor H5 (ITIH5), transcript variant 1, mRNA [NM_030569]

					**up**	MCHR1	NM_005297	melanin-concentrating hormone receptor 1 (MCHR1), mRNA [NM_005297]

					**up**	MGAT4A	NM_012214	mannosyl (alpha-1,3-)-glycoprotein beta-1,4-N-acetylglucosaminyltransferase, isozyme A (MGAT4A), mRNA [NM_012214]

					**up**	GPR65	NM_003608	G protein-coupled receptor 65 (GPR65), mRNA [NM_003608]

					**up**	DFNB31	AK056190	cDNA FLJ31628 fis, clone NT2RI2003344, weakly similar to PRESYNAPTIC PROTEIN SAP97. [AK056190]

					**up**	LRP8	NM_033300	low density lipoprotein receptor-related protein 8, apolipoprotein e receptor (LRP8), transcript variant 2, mRNA [NM_033300]

*dn*		**up**	**up**		**up**	SORCS3	NM_014978	sortilin-related VPS10 domain containing receptor 3 (SORCS3), mRNA [NM_014978]

*dn*		**up**				HLA-DPA1	NM_033554	major histocompatibility complex, class II, DP alpha 1 (HLA-DPA1), mRNA [NM_033554]

		**up**	*dn*	*dn*		AKR1C1	BC040210	aldo-keto reductase family 1, member C1, mRNA (cDNA clone MGC:42600 IMAGE:4825338), complete cds. [BC040210]

		**up**		*dn*	**up**	WDR64	NM_144625	WD repeat domain 64 (WDR64), mRNA [NM_144625]

		*dn*			**up**	ESCO2	NM_001017420	establishment of cohesion 1 homolog 2 (S. cerevisiae) (ESCO2), mRNA [NM_001017420]

		*dn*			**up**	CACNG5	NM_145811	calcium channel, voltage-dependent, gamma subunit 5 (CACNG5), transcript variant 1, mRNA [NM_145811]

		*dn*			**up**	ANPEP	NM_001150	alanyl (membrane) aminopeptidase (aminopeptidase N, aminopeptidase M, microsomal aminopeptidase, CD13, p150) (ANPEP), mRNA [NM_001150]

				*dn*	**up**	GPX7	NM_015696	glutathione peroxidase 7 (GPX7), mRNA [NM_015696]

			*dn*	*dn*	**up**	TTLL7	NM_024686	tubulin tyrosine ligase-like family, member 7 (TTLL7), mRNA [NM_024686]

			*dn*	*dn*	**up**	THC2371963	THC2371963	AIP1_HUMAN (Q86UL8) Atrophin-1 interacting protein 1 (Atrophin-1 interacting protein A) (MAGI-2), partial (3%) [THC2371963]

	**up**			*dn*	*dn*	GDF15	NM_004864	growth differentiation factor 15 (GDF15), mRNA [NM_004864]

				*dn*	*dn*	ALDH1A1	NM_000689	aldehyde dehydrogenase 1 family, member A1 (ALDH1A1), mRNA [NM_000689]

				*dn*	*dn*	DCN	NM_001920	decorin (DCN), transcript variant A1, mRNA [NM_001920]

				*dn*	*dn*	SOX2	NM_003106	SRY (sex determining region Y)-box 2 (SOX2), mRNA [NM_003106]

				*dn*	*dn*	TAF9B	NM_015975	TAF9B RNA polymerase II, TATA box binding protein (TBP)-associated factor, 31 kDa (TAF9B), mRNA [NM_015975]

				*dn*	*dn*	THC2302184	THC2302184	GAL2_HUMAN (Q01415) N-acetylgalactosamine kinase (GalNAc kinase) (Galactokinase 2), partial (21%) [THC2302184]

				*dn*	*dn*	PTPRZ1	NM_002851	protein tyrosine phosphatase, receptor-type, Z polypeptide 1 (PTPRZ1), mRNA [NM_002851]

				*dn*	*dn*	DMRT2	NM_006557	doublesex and mab-3 related transcription factor 2 (DMRT2), transcript variant 1, mRNA [NM_006557]

			*dn*	*dn*		CLEC3A	NM_005752	C-type lectin domain family 3, member A (CLEC3A), mRNA [NM_005752]

			*dn*	*dn*		ATP10B	AB018258	mRNA for KIAA0715 protein, partial cds. [AB018258]

			*dn*	*dn*		PROS1	NM_000313	protein S (alpha) (PROS1), mRNA [NM_000313]

			*dn*	*dn*		RAXLX	NM_001008494	RAX-like homeobox (RAXLX), mRNA [NM_001008494]

			*dn*	*dn*		OLFM4	NM_006418	olfactomedin 4 (OLFM4), mRNA [NM_006418]

			*dn*	*dn*		DSG4	NM_177986	desmoglein 4 (DSG4), mRNA [NM_177986]

			*dn*	*dn*	*dn*	IQGAP2	NM_006633	IQ motif containing GTPase activating protein 2 (IQGAP2), mRNA [NM_006633]

			*dn*	*dn*	*dn*	MAL	NM_002371	mal, T-cell differentiation protein (MAL), transcript variant a, mRNA [NM_002371]

			*dn*	*dn*	*dn*	KRT1	NM_006121	keratin 1 (epidermolytic hyperkeratosis) (KRT1), mRNA [NM_006121]

			*dn*	*dn*	*dn*	MMP1	NM_002421	matrix metallopeptidase 1 (interstitial collagenase) (MMP1), mRNA [NM_002421]

		*dn*		*dn*	*dn*	LAMP3	NM_014398	lysosomal-associated membrane protein 3 (LAMP3), mRNA [NM_014398]

		*dn*		*dn*	*dn*	HLA-DMB	NM_002118	major histocompatibility complex, class II, DM beta (HLA-DMB), mRNA [NM_002118]

		*dn*		*dn*	*dn*	HERC5	NM_016323	hect domain and RLD 5 (HERC5), mRNA [NM_016323]

					*dn*	BTN3A3	NM_006994	butyrophilin, subfamily 3, member A3 (BTN3A3), transcript variant 1, mRNA [NM_006994]

					*dn*	C6orf130	NM_145063	chromosome 6 open reading frame 130 (C6orf130), mRNA [NM_145063]

					*dn*	CRY2	NM_021117	cryptochrome 2 (photolyase-like) (CRY2), mRNA [NM_021117]

					*dn*	TMEM140	NM_018295	transmembrane protein 140 (TMEM140), mRNA [NM_018295]

					*dn*	ZNF438	NM_182755	zinc finger protein 438 (ZNF438), mRNA [NM_182755]

					*dn*	DNMT3L	NM_013369	DNA (cytosine-5-)-methyltransferase 3-like (DNMT3L), transcript vari ant 1, mRNA [NM_013369]

		*dn*				CTAGE3	AF338231	CTAGE-3 protein mRNA, complete cds. [AF338231]

		*dn*				MOSPD2	NM_152581	motile sperm domain containing 2 (MOSPD2), mRNA [NM_152581]

		*dn*				OR4N4	NM_001005241	olfactory receptor, family 4, subfamily N, member 4 (OR4N4), mRNA [NM_001005241]

		*dn*				SPATA7	NM_018418	spermatogenesis associated 7 (SPATA7), transcript variant 1, mRNA [NM_018418]

		*dn*				TMC1	NM_138691	transmembrane channel-like 1 (TMC1), mRNA [NM_138691]

^# ^listed genes were statistically significant different and exhibited at least a 5fold induction or repression in one of the treatments (with respect to controls and based on normalised fluorescence intensity ratios in the microarray analysis); type of gene regulation is indicated as "up" for induction (> 2fold) and "dn" for repression (> 2fold); fields are empty when induction or repression was < 2fold; full table is provided as Additional File 1

### Confirmation of microarray data

**Figure 1:**
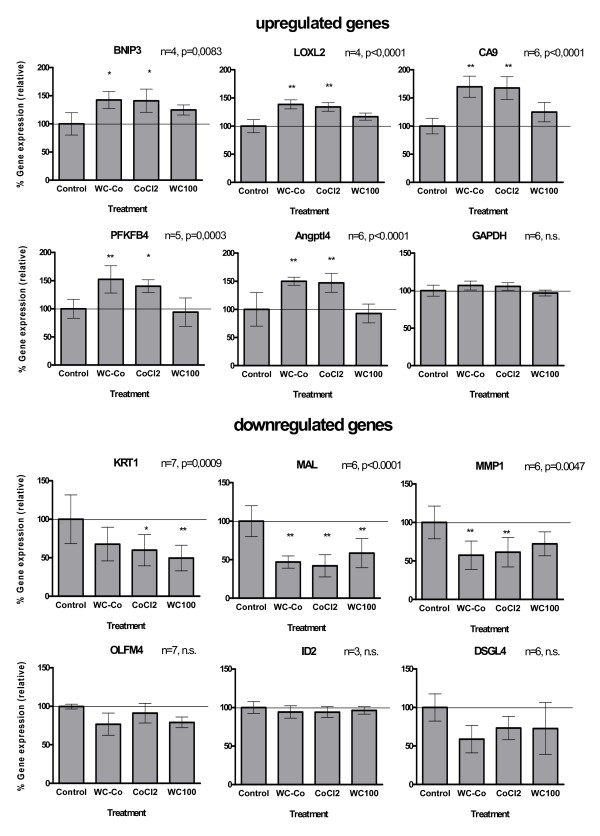
**Validation of microarray data by RT-PCR**. Relative gene expression of arbitrarily selected genes in HaCaT cells after 3 d of exposure to 30 μg/ml WC and 33 μg/ml WC-Co nanoparticles and 3 μg/ml CoCl_2 _was analysed by semiquantiative RT-PCR. Selected genes represent genes with significant changes (2 to 23fold) of expression levels in microarrays. Gene expression values were converted to percent of the mean of controls and are presented as mean + standard deviation (SD). Statistical differences were analysed with one-way ANOVA followed by Dunnett's post test (treatment vs. control). Values of p < 0.05 were considered statistically significant; *p < 0.05, **p < 0.01.

In order to verify the microarray results, RT-PCR analysis was conducted using the same set of samples used for the microarrays as well as RNA samples from independent exposure experiments. Twelve genes with significantly differential expression and a minimum of 2fold up- or downregulation were selected for RT-PCR confirmation. However, care was taken that genes with weak (close to 2fold differential expression) and strong changes (up to 23fold) in expression were included (Figure 1). For eight of the selected genes the significant changes of expression could be confirmed (BNIP3, LOXL2, ANGPTL4, CA9, PFKFB4, KRT1, MAL, MMP1). Trends (induction or repression) were conserved between microarray and RT-PCR data for each treatment. The remaining genes (GAPDH, ID2, OLFM4, DSG4) exhibited a high variability and could not be confirmed as statistically significant from controls by RT-PCR.

### PCA and cluster analyses

**Figure 2:**
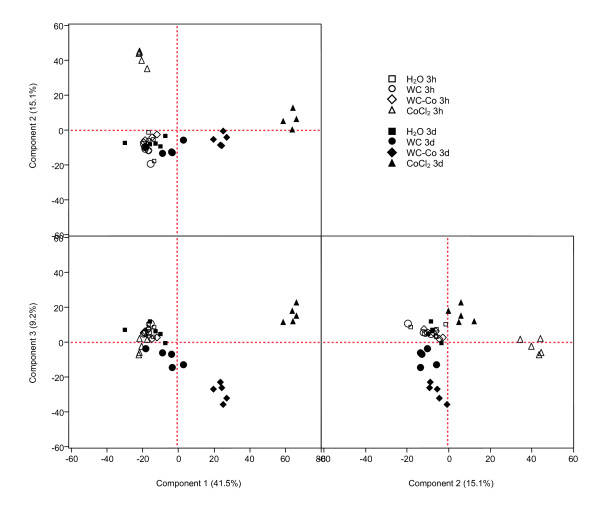
**Principle component analyses**. PCA of differentially expressed genes in HaCaT cells exposed for 3 h and 3 d to 30 μg/ml WC, 33 μg/ml WC-Co nanoparticles and 3 μg/ml CoCl_2_. Each symbol represents a biological replicate.

Two methods of descriptive statistics - PCA (principal component analysis) and HCA (hierarchical cluster analysis) - were applied to identify commonalities or differences between treatments based on the patterns of significantly differentially expressed genes. By PCA analysis about 65% of the variability in different treatments was represented by the first 3 components. Four clearly separated clusters, i.e. cells treated with CoCl_2 _for 3 h, the same treatment for 3 d, cells treated with WC-Co for 3 d and cells treated with WC for 3 d were identified (Figure 2). All other treatments, including the controls, were not separated and formed a large cluster with apparently weak gene expression changes if compared to controls. WC-treated cells were less clearly separated from controls. This was indicated by the observation that a clear distinct cluster was only demonstrated for PC axis 2 and 3.

Similar results were obtained by HCA, which identified 3 treatment clusters. Both CoCl_2 _treatments and the 3 d exposures of WC-Co formed a distinct cluster. All other treatments (controls, WC3d, WC3h, WC-Co3h) were grouped in one cluster (Additional file 2). Gene clustering revealed two clusters with strongly induced genes. Genes of these clusters (i.e. LOXL2, BNIP3, CA9, PDK1, ASB2, EGLN3, ANKRD37, PNCK) are coding for proteins with diverse functions, but some of them are known to be direct targets of the transcription factor HIF1α (see below). For the remaining clusters it was not possible to identify predominating groups of gene ontology. Therefore, gene clusters were not analysed in detail but two types of pathway analysis were used to identify signalling pathways and biological functions associated with the differentially expressed genes.

### Gene set enrichment analysis (GSEA) and identification of affected signalling pathways

Pathway analysis was performed by two approaches, with (1) GSEA software [34,35] using the complete set of gene expression data from the microarray experiments, and (2) the DAVID database [36] using the list of more then 2fold differentially expressed genes previously identified with SAM.

GSEA identified communalities with existing gene sets (enrichment) primarily for induced genes. The highest number of affected gene sets was found for the 3 d WC-Co treatment. The majority of pathways associated with down-regulated gene sets were found after 3 h of exposure with WC-Co. An overview of pathway related gene sets with the highest commonality (based on statistical significance analysis) to the observed patterns of differentially expressed genes is given in Table 3.

**Table 3:** Gene sets with strongest overlaps with observed differentially expressed genes.*

up-regulated gene sets	description	WC 3 h	WCCo 3 h	CoCl2 3 h	WC 3 d	WCCo 3 d	CoCl2 3 d

***hypoxia related gene sets***							

HIF1_TARGETS	Hif-1 (hypoxia-inducible factor 1) transcriptional targets		0.17			0.01	

HIFPATHWAY	BIOCARTA: Under normal conditions, HIF-1 is degraded; under hypoxic conditions, it activates transcription of genes controlled by hypoxic response elements (HREs)					0.02	0.20

HYPOXIA_FIBRO_UP	Up-regulated by hypoxia in normal fibroblasts from both young and old donors		0.20		0.65	0.05	

HYPOXIA_NORMAL_UP	Up-regulated by hypoxia in normal, RPTEC renal cells		0.50			0.02	

HYPOXIA_REG_UP	Up-regulated by hypoxia in renal cells, and down-regulated with reoxygenation		0.05			0.01	0.03

HYPOXIA_REVIEW	Genes known to be induced by hypoxia		0.22		0.75	0.00	

MANALO_HYPOXIA_UP	Genes up-regulated in human pulmonary endothelial cells under hypoxic conditions or after exposure to AdCA5, an adenovirus carrying constitutively active hypoxia-inducible factor 1 (HIF-1alpha)		0.24			0.01	0.21

MENSE_HYPOXIA_UP	List of Hypoxia-induced genes found in both Astrocytes and HeLa Cell		0.00			0.02	0.13

RESPONSE_TO_HYPOXIA	GO:0001666. Change in state or activity of a cell or an organism (in terms of movement, secretion, enzyme production, gene expression, etc.) as a result of a stimulus indicating lowered oxygen tension					0.12	0.08

***carbohydrate metabolism***							

POLYSACCHARIDE_METABOLIC_PROCESS	GO:0005976. Chemical reactions and pathways involving polysaccharides, a polymer of more than 10 monosaccharide residues joined by glycosidic linkages					0.06	

FRUCTOSE_AND_MANNOSE_METABOLISM	Genes involved in fructose and mannose metabolism		0.21			0.02	

HSA00010_GLYCOLYSIS_AND_ GLUCONEOGENESIS	KEGG: Genes involved in glycolysis and gluconeogenesis					0.00	0.14

GLYCOGEN_METABOLISM	Genes involved in glycogen metabolism					0.04	

GALACTOSE_METABOLISM	Genes involved in galactose metabolism					0.04	

PENTOSE_PHOSPHATE_PATHWAY	Genes involved in pentose phosphate pathway				0.61	0.14	

STARCH_AND_SUCROSE_ METABOLISM	Genes involved in starch and sucrose metabolism					0.01	0.50

***endocrine metabolism***							

GN_CAMP_GRANULOSA_UP	Up-regulated in human granulosa cells by the gonadotropins LH and FSH, as well as by cAMP- stimulator forskolin					0.01	0.17

LH_GRANULOSA_UP	Up-regulated in human granulosa cells stimulated with luteinizing hormone (LH)					0.01	

FSH_GRANULOSA_UP	Up-regulated in human granulosa cells stimulated with follicle stimulation hormone (FSH)					0.01	

BREAST_CANCER_ESTROGEN_SIGNALING	Genes preferentially expressed in breast cancers, especially those involved in estrogen-receptor- dependent signal transduction					0.05	

PROSTAGLANDIN_SYNTHESIS_ REGULATION	WIKIPATHWAYS: Prostaglandin Synthesis and Regulation		0.09				

HSA04150_MTOR_SIGNALING_ PATHWAY	KEGG: Genes involved in mTOR signalling pathway		0.19				

***cell adhesion, structure, cytoskeleton***							

HSA04510_FOCAL_ADHESION	KEGG: Genes involved in focal adhesion					0.07	0.46

ACTIN_CYTOSKELETON	GO:0015629. Part of the cytoskeleton (the internal framework of a cell) composed of actin and associated proteins					0.04	

ACTIN_BINDING	GO:0003779. Interacting selectively with monomeric or multimeric forms of actin, including actin Filaments					0.06	

CYTOSKELETON_DEPENDENT_ INTRACELLULAR_TRANSPORT	GO:0030705. The directed movement of substances along cytoskeletal elements such as microfilaments or microtubules within a cell					0.07	

ANATOMICAL_STRUCTURE_FORMATION	GO:0048646. Process pertaining to the initial formation of an anatomical structure from unspecified parts					0.04	

VASCULATURE_DEVELOPMENT	GO:0001944. Process whose specific outcome is the progression of the vasculature over time, from its formation to the mature structure					0.04	

ANGIOGENESIS	GO:0001525. Blood vessel formation when new vessels emerge from the proliferation of pre-existing blood vessels					0.05	

HSA04512_ECM_ RECEPTOR_INTERACTION	KEGG: Genes involved in ECM-receptor interaction					0.06	0.29

***miscellaneous***							

G13_SIGNALING_PATHWAY	G13 signaling pathway					0.10	0.51

NUCLEOTIDE_BIOSYNTHETIC_PROCESS	GO:0009165. Chemical reactions and pathways resulting in the formation of nucleotides					0.09	

CARBON_CARBON_LYASE_ACTIVITY	GO:0016830. Catalysis of the cleavage of C-C bonds by other means than by hydrolysis or oxidation, or conversely adding a group to a double bond					0.10	

ALKPATHWAY	Activin receptor-like kinase 3 (ALK3) is required during gestation for cardiac muscle development						0.29

CARDIACEGFPATHWAY	BIOCARTA: Cardiac hypertrophy, a response to high blood pressure, is stimulated by GPCR ligands such as angiotensin II that activate the EGF pathway		0.21			0.01	

WNT_SIGNALING	Wnt signaling genes					0.01	

HSA05211_RENAL_CELL_CARCINOMA	Genes involved in renal cell carcinoma					0.07	

NKTPATHWAY	BIOCARTA: T cell differentiation into Th1 and Th2 cells occurs by differential chemokine receptor expression, which mediates tissue localization and immune response						0.29

BIOGENIC_AMINE_SYNTHESIS	WIKIPATHWAYS: Genes involved in synthesis of biogenic amines		0.23				

HSA00591_LINOLEIC_ACID_METABOLISM	Genes involved in linoleic acid metabolism		0.22				

HSA00361_GAMMA_HEXACHLOROCYCLOHEXANE_DEGRADATION	KEGG: Genes involved in gamma-hexachlorocyclohexane degradation		0.17				

**down-regulated gene sets**	**description**	**WC 3 h**	**WCCo 3 h**	**CoCl2 3 h**	**WC 3 d**	**WCCo 3 d**	**CoCl2 3 d**

***RNA metabolism and processing***							

MRNA_METABOLIC_PROCESS	GO:0016071. Chemical reactions and pathways involving mRNA		0.23				

RIBONUCLEOPROTEIN_COMPLEX_	GO:0022613. The cellular process by which a complex containing RNA and proteins, is synthesized,		0.25				

BIOGENESIS_AND_ASSEMBLY	aggregates, and bonds together						

RNA_PROCESSING	GO:0006396. Any process involved in the conversion of one or more primary RNA transcripts into one or more mature RNA molecules		0.20				

RNA_SPLICING__VIA_TRANSESTERIFICATION_ REACTIONS	GO:0000375. Splicing of RNA via a series of two transesterification reactions		0.20				

SEQUENCE_SPECIFIC_ DNA_BINDING	GO:0043565. Interacting selectively with DNA of a specific nucleotide composition, e.g. GC-rich DNA binding, or with a specific sequence motif or type of DNA e.g. promotor binding or rDNA binding		0.33				

TRNA_METABOLIC_PROCESS	GO:0006399. Chemical reactions and pathways involving tRNA		0.27				

***nucleus and the nuclear membrane related gene sets***							

PORE_COMPLEX	GO:0046930. Any small opening in a membrane that allows the passage of gases and/or liquids.		0.28				

NUCLEAR_PORE	GO:0005643. Any of the numerous similar discrete openings in the nuclear envelope of a eukaryotic cell, where the inner and outer nuclear membranes are joined		0.19				

NUCLEAR_LUMEN	GO:0031981. The volume enclosed by the nuclear inner membrane		0.31				

NUCLEAR_MEMBRANE	GO:0031965. Either of the lipid bilayers that surround the nucleus and form the nuclear envelope; excludes the intermembrane space		0.31				

***enzyme and receptor activity***							

UBIQUITIN_PROTEIN_ LIGASE_ACTIVITY	GO:0004842. Catalysis of the reaction: ATP + ubiquitin + protein lysine = AMP + diphosphate + protein N-ubiquityllysine		0.32				

SMALL_PROTEIN_CONJUGATING_ ENZYME_ACTIVITY	GO:0008639. Catalysis of the covalent attachment of small proteins, such as ubiquitin or ubiquitin-like proteins, to lysine residues on a target protein. This function may be performed alone or in conjunction with an E3, ubiquitin-like protein ligase		0.21				

CASPASEPATHWAY	BIOCARTA: Caspases are cysteine proteases active in apoptosis				0.30		

* identified by GSEA (gene set enrichment analysis), using the databases MSigDB C2 and C5; number of entries is limited to gene sets with a false discovery rate (FDR) < 0.35 in at least one of the treatments and FDR < 0.1 for the WC-Co 3 d treatment; only gene sets obviously related to biochemical pathways or cellular organelles were selected; full table provided as Additional File 3

Gene sets related to the hypoxia pathway as well as carbohydrate metabolism were induced by WC-Co and CoCl_2 _after 3 d. A significant association with the induction of the hypoxia gene sets was also observed after 3 h of exposure with WC-Co. As indicated by the enrichment of genes for the transcription factor HIF1α (hypoxia inducible factor 1 alpha), regulation via HIF1α may play a major role in provoking the observed changes in hypoxia and carbohydrate metabolism genes. Furthermore, GSEA detected an enrichment of genes related to RNA metabolism and processing as well as genes coding for proteins of the nucleus and the nuclear membrane. These gene sets referred mainly to genes down-regulated after 3 h of exposure to WC-Co nanoparticles. Some of the genes with strong differential repression (> 5fold; e.g. MAL, KRT1, GDF15, MMP1; identified by SAM) were not found to be included in these pathways.

DAVID revealed similar results as GSEA. However, small gene sets, for instance a down-regulation of metallothioneins in the 3 h CoCl_2 _exposure, were additionally identified by DAVID. Furthermore, genes coding for several proteins containing a functional prolyl-4-hydroxylase alpha subunit were highlighted as up-regulated for the 3 d of exposure with CoCl_2 _and WC-Co.

The complete results of the GSEA and DAVID analyses are provided in the Additional file 3.

## Discussion

The increasing use of nanoparticles may also lead to an increased human exposure and adverse health effects. Occupational exposure is one of the most relevant exposure routes. In order to estimate the potential human health impacts of nanoparticles a precise knowledge on their mechanism of action is indispensable. This knowledge allows, for instance, clarifying whether effects are specifically associated with or enhanced by the nano-sized dimensions or whether the same type of effects as known for corresponding bulk material or dissolved compounds occurs.

In the present paper we focussed on WC and WC-Co nanoparticles which are used in hard metal industries. Dermal uptake, inhalation or accidental oral uptake present possible routes for occupational exposure for these particles. Our previous study has demonstrated their incorporation into various types of cells. Toxicity was low but enhanced for WC-Co compared to pure WC particles [21,22]. A transcriptome analysis of human macrophages exposed to μm-sized WC-Co revealed differential expression of genes known to be affected by cobalt as well [33], providing first evidence that dissolved cobalt seems to play a role in WC-Co toxicity. However, no direct comparison of transcription patterns provoked by nano-sized WC-Co, WC and dissolved cobalt is available so far. In order to model human skin exposure, the human keratinocyte cell line (HaCaT) was selected as experimental model to perform microarray analyses. A number of statistical methods and database analysis tools were used to compare the data sets and perform a detailed pathway analysis.

### Transcriptional changes in WC exposed cells

Identification of significantly altered genes revealed only little changes for the exposure of HaCaT cells to WC. Similar observations were made for WC in larger particle size *in vitro *and *in vivo *[16,19]. The weak transcriptomic response may be explained by the physicochemical characteristics, since WC nanoparticles were shown to be chemically inert [21]. The genes detected as differentially expressed with WC were mostly also affected by WC-Co and CoCl_2 _(e.g. EGLN3, CA9, BNIP3, LOXL2, PDK1, KRT1, MMP1). This might be due to traces of cobalt and other metals in WC nanoparticle preparations that have been reported at low concentrations of about 5 × 10^-4 ^μM (described by Bastian et al., 2009 [21]). Some of the genes, however, showed a reciprocal differential expression pattern. For example, while induced by CoCl_2_, a repression was detected for WC and WC-Co nanoparticles, for e.g. TTLL7, KIT, CHST6, NODAL, WDR64, DES, HS6ST3, DLX2, GPR158. In order to identify potential effects associated with the dimensions of nanoparticles but not related to the chemical compound, we compared our expression data set with 503 genes that were found to be affected by exposure to amorphous silica nanoparticles [12]. In this study, transcriptomic profile of macrophages exposed to amorphous silica particles in two different sizes and different concentrations were recorded. Similar to WC, amorphous silica is known to be chemically inert. Only 29 out of 503 of the silica-sensitive genes were also found to be differentially expressed in our study. Since these genes showed an altered expression with CoCl_2 _rather than with WC particles, they may reflect a general unspecific stress response.

### Transcriptional changes in WC-Co and CoCl_2 _exposed cells

Compared to the effects with WC nanoparticles, more genes were affected by the WC-Co nanoparticles. Most of them were altered by CoCl_2 _as well. We found strong overlaps of the expression data of WC-Co and CoCl_2 _treated cells, whereas the highest number of genes differentially expressed was found with CoCl_2_. As demonstrated by GSEA analysis, the differentially expressed genes involved in the transcriptional response to WC-Co and CoCl_2 _could be associated to various biological functions or signalling pathways which are discussed in detail in the following paragraphs.

Whereas most of the affected genes were induced, a number of genes repressed by WC, WC-Co and CoCl_2 _exposure have been found as well (e.g. MAL, OLFM4, KRT1, CLCA2, MMP1, IQGAP2). For most of these genes the mechanisms of transcriptional regulation are not known and special pathways related to this group of genes could not be identified.

### The role of HIF1 for differential gene expression in WC-Co and CoCl_2 _treated cells

**Figure 3:**
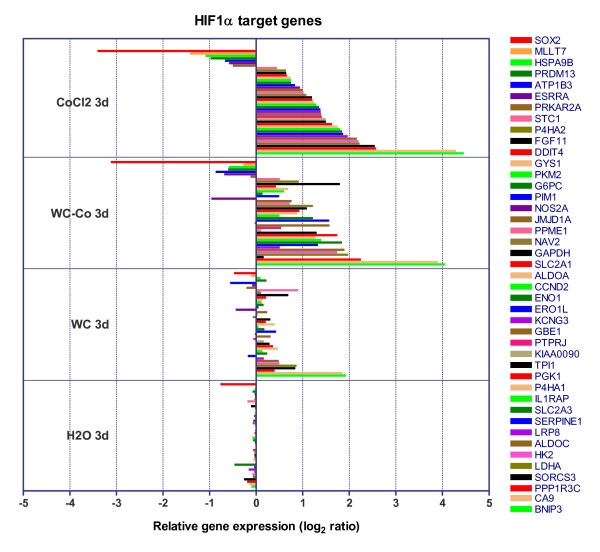
**Expression of HIF1α target genes**. HIF1α target genes and their expression levels after 3 d of exposure of HaCaT cells to 30 μg/ml WC and 33 μg/ml WC-Co nanoparticles and 3 μg/ml CoCl_2_. Bars indicate the mean microarray expression levels of 5 biological replicates. This figure represents all affected HIF target genes identified as significantly differentially expressed by SAM without a fold change threshold.

Comparison of the pattern of significant genes and gene set enrichment analyses demonstrated similar responses and signalling pathways for cells exposed to WC-Co and CoCl_2_, e.g. genes involved in the metabolism of glycolysis and gluconeogenesis, cell adhesion and the response to hypoxia. Under hypoxic conditions, the α subunit of hypoxia inducible factor 1 (HIF1α) accumulates and induces transcription of diverse target genes. HIF1α is a transcription factor that is ubiquitously expressed but rapidly degraded under normoxic conditions. Cobalt ions are known to stabilise HIF1α under normoxic conditions and therefore exert hypoxia-like cellular responses [24-26,37]. Several genes sorted into gene sets related to hypoxia and other pathways e.g. glycolysis and gluconeogenesis, are primary targets of the transcription factor HIF1. To analyse whether such HIF1 target genes were affected by our treatments we generated a list of HIF1target genes (list contained two gene sets from the GSEA C3 TFT database and the "HIF1_Targets" gene set (C2) that was generated after Semenza (2001) [28]). When the expression of HIF1 primary targets is compared, WC-Co nanoparticles provoke almost the same pattern of induction or repression as CoCl_2 _(Figure 3).

### HIF1 as an initial factor for downstream regulation

The list of affected HIF1 targets contained transcription factors that could be involved in the regulation of secondary HIF1 targets. One example is SOX2. SOX2 contains a putative HIF1 promotor binding site and was found to be strongly repressed after 3 d of exposure with WC-Co and CoCl_2_. SOX2 is known to play a key role in stem cell generation and pluripotency [38-40]. Greber et al. (2007) [41] studied the transcription profile of embryonic stem cells and embryonic carcinoma cells following a knock down of SOX2. In HaCaT cells exposed to CoCl_2_, 97 genes with differential expression (26 repressed, 71 induced genes) showed a similar expression pattern if compared to the SOX2 knock down. None of these genes is known as direct HIF1 targets or exhibit HIF1 binding sites and quite a few of them were mentioned in the context of hypoxia earlier. These genes might be regulated by the SOX2 transcription factor as potential secondary HIF1 targets.

Endothelin 2 (EDN2) is another example for a gene with a putative promoter HIF1 binding site that could be involved in the differential expression of genes in the CoCl_2 _treatment. In agreement with the HIF1 promotor binding site, Na et al. (2008) [42] reported the induction of EDN2 after 3 and 6 hours of hypoxic treatment in granulosa cells. Similarly, EDN2 was found to be induced in HaCaT cells exposed to WC-Co and CoCl_2 _after 3 h. An induction of other genes of the endothelin complex (EDNRB, EDNRA, ECE2) was detected with the same treatments after 3 d. The induction of collagen mRNA levels and the repression of the matrix metallopeptidase 1 (MMP1) by the endothelin complex was described by Shi-Wen and colleagues [43]. Indeed, an induction of the collagen gene COL5A1 and the repression of the endothelin downstream target MMP1 were detected in HaCaT cells exposed to WC-Co and CoCl_2 _for 3 d.

In addition to the sets of genes regulated by HIF1, GSEA identified sets of genes that are regulated by other transcription factors. However, similar to HIF1, transcripts of the genes encoding the transcription factors themselves were not found to be differentially expressed. The list (see Additional file 3) contained transcription factors known to be HIF1α interaction partners - e.g. ARNT (aryl hydrocarbon nuclear translocator, [44]) - or HIF1 supporting factors - e.g. (AP1, [45]; Smad3/4; [46]), but also a number of the enriched gene sets not known to be related to HIF1 or targets of HIF1 (such as BACH2, NEF2, ALX4, PAX3).

By comparing HIF1 target genes with known hypoxia responsive genes it becomes obvious that only part of the hypoxia related genes are known to be direct or indirect targets of HIF1. Nevertheless, the fact that most of the genes and pathways affected with WC-Co and CoCl_2 _were also observed in toxicogenomic studies investigating hypoxia [8,47,48] led us to conclude that the stabilisation of HIF1α via cobalt is an initial step and most of the reactions that are not directly related to HIF1α might reflect downstream events.

### Cobalt ions as co-factor substitute

In CoCl_2 _treated cells the YY1 transcription factor was identified as a potential master regulating factor with GSEA. YY1 is a ubiquitous transcription factor with fundamental biological functions. Its role in cancer biology is also intensely discussed [49]. An interaction of YY1 with cobalt was not yet described but might be conceivable, because YY1 contains four zinc finger domains. The substitution of zinc ions and other divalent metal ions by Co^2+ ^is often discussed to play a role in transcription factor domains, DNA repair mechanisms and calcium metabolism [50-52]. The transcriptional changes of YY1 target genes after the CoCl_2 _exposure indicate an induction or enrichment of the YY1 protein but it remains unclear, whether the substitution of zinc ions by Co^2+ ^is responsible for that.

In HaCaT cells, a significant depletion of intracellular Zn^2+ ^and Mg^2+ ^after CoCl_2 _exposure was described by Ortega and colleagues [53]. A substitution of magnesia ions by Co^2+ ^may result in the interruption of ATPases and the energy balance of the cell [54]. It is proposed that ion substitution plays a role in uptake mechanisms of cobalt ions into cells, which evidently happens via cation-dependent ionic pumps [50,53]. Although we found gene sets related to metal ion or cation ion binding proteins to be affected, we did not detect an enrichment of gene sets connected to the described effects resulting from ion substitution.

### Differential expression of protein kinases and phosphatases

It was noticed that in WC-Co and CoCl_2 _exposures several kinases and phosphatases exhibited a differential expression. Kinases are a major group of proteins involved in endocytosis [55,56]. In a knock-down study of Pelkmans and colleagues (2005) [56] 209 kinases with known important functions in several pathways were identified to be involved in endocytosis. Interestingly, most of the pathways they could link with endocytotic processes were also affected in our study (e.g. mTOR, Wnt, integrin/adhesion, RTKs/RSTKs, GPCR).

Protein tyrosine phosphatases (PTPs) and kinases as well as enzymes of the phosphoinositol-3-kinase family (PI3Ks) seem to play a special role in the response to cobalt. The latter have been described as possible upstream regulators of HIF1α [57-60] and have functions in some of the pathways found to be induced (e.g. Insulin-, IGF1-, PPARα-pathway; based on GSEA analysis). PTPs are known to be signalling molecules that regulate a variety of cellular processes including cell growth, differentiation, mitotic cycle, and oncogenic transformation. Some genes coding for PTPs were not only affected by the cobalt containing treatments but also by WC (PPFIA4, PTPRT, PTPRZ1). Since kinases and phosphatases are also involved in the cellular response to various kinds of environmental stress, their altered expression may be related to a cobalt-induced and/or a particle uptake related stress response.

### Oxidative stress and transcriptional response

The production of reactive oxygen species (ROS) and the subsequent induction of oxidative stress are discussed as major modes of action of nanoparticles [2,61,62] and was also described to be involved in the cellular response to cobalt ions [54,63,64]. Nevertheless, genes or gene clusters that are related to oxidative stress responses could not be found within our data set of differentially expressed genes. This was confirmed by a lack of ROS production in HaCaT cells for any of the treatments (manuscript in preparation).

### Cobalt ions and WC-Co toxicity

Cobalt is an essential trace element for humans, but becomes toxic at high concentrations. In a previous study, we analysed acute toxicity by measuring cell viability of HaCaT cells after same exposure conditions as performed in this study [21]. Altered proliferation or morphological changes of the cells were not observed. Toxicity of CoCl_2 _was indicated by decreasing cell viability at concentration of 100 μM (corresponds to 6 μg/ml) and above. Lower concentrations of CoCl_2 _have not been observed to cause acute toxicological reactions in several cells *in vitro*, including in HaCaT cells [21,54,65]. Intense transcriptional changes were observed in this study at concentrations slightly below those causing *in vitro *toxicity. The differentially expressed genes may serve as indicators for potential long term effects and may also be useful for investigations of molecular mechanisms.

WC-Co nanoparticles exhibited an increased toxicity in previous studies performed in different types of cell lines (human and fish) when compared to WC particles and CoCl_2 _[21,22]. Viability of HaCaT cells was slightly (15%) decreased after 3 days of exposure. This enhanced toxicity was discussed as either a result of increased cellular cobalt uptake associated with the uptake of WC particles - the so called "Trojan horse" hypothesis [61] - or a result of unknown combinatory effects of WC particles and cobalt. The "trojan horse" theory is supported by studies showing increased toxicity of nanoparticles with leaching ions compared to the ions alone [11,66-68]. However, analysing the global transcriptional response of HaCaT cells to WC-Co nanoparticles and equivalent WC and cobalt treatments, no evidence for either of these theories could be provided. The number of transcriptional changes was more pronounced in CoCl_2 _exposed cells, but particularly the regulation of genes resulting from cobalt dependent stabilisation of HIF1α was similar for both, WC-Co and CoCl_2_. The patterns of transcriptional regulation clearly indicate that the majority of the effects were associated with cobalt ions and did not indicate a special type of interaction between WC and cobalt. However, the enhanced toxicity of WC-Co with respect to CoCl_2 _appears to be mediated via unknown non-transcriptionally regulated pathways.

## Conclusion

**Figure 4:**
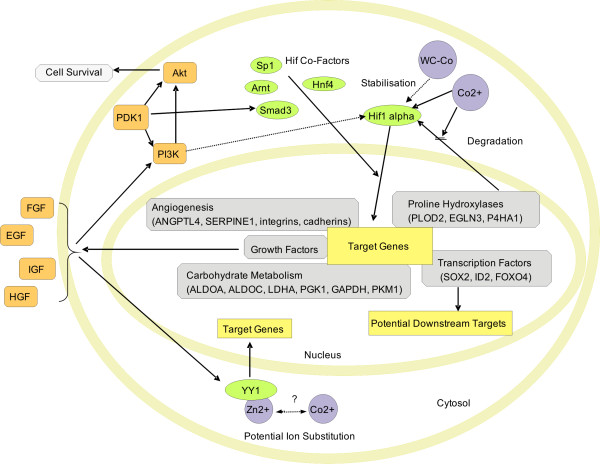
**Scheme of affected cellular pathways**. Illustration of the major cellular signalling pathways that were indicated by analyses of the transcriptional responses to WC-Co nanoparticles and cobalt chloride. Arrows indicate known (full lines) or potential (dashed lines) interactions. (Complex Proteins = orange, Transcription Factors = green).

Analysis of gene expression patterns in the human keratinocyte cell line HaCaT demonstrated that the transcriptional response to WC-Co nanoparticles is mainly caused by cobalt ions leaching from the particles. While WC nanoparticles alone do only show very weak effects in expression patterns, WC-Co and CoCl_2 _exhibited significant transcriptional changes in genes involved in carbohydrate metabolism, hypoxia response, endocrine pathways, cell adhesion and others. The cobalt-sensitive transcription factor HIF1 plays an important role in the regulation of genes involved in these pathways, showing that WC-Co nanoparticles exert hypoxia-like responses similar to CoCl_2_. The subacute response to CoCl_2 _was analysed and discussed with respect to downstream events of HIF1 and involvement of other transcription factors (e.g. SOX2, YY1) in cobalt toxicity. A simplified scheme of potential major pathways resulting from cobalt reactions within the cell is provided in Figure 4.

However, while detailed analyses of transcriptional regulations clearly indicate that leached cobalt is likely to be the major trigger for gene regulation in cells exposed to WC-Co, the changes in transcription patterns do not explain the enhanced toxicity of WC-Co if compared to equivalent concentrations of WC or CoCl_2_. This enhanced toxicity is suggested to be mediated by unknown combinatory effects of WC and cobalt not reflected primarily at the transcriptional response level. However, support or rejection of this hypothesis requires further investigations.

## Methods

### Preparation and characterisation of particle suspensions and cobalt chloride solutions

Particles and exposure conditions used in this study were identical to those in a previous study which also describes details of the particle preparation, characterisation and behaviour in cell culture media [21]. Briefly, particle suspensions with a concentration of 100 μg/ml were prepared from tungsten carbide (WC) and tungsten carbide cobalt (WC-Co; 10 mass % cobalt content) powders as described in detail by Bastian et al. [21]. Particles exhibited a Brunauer-Emmett-Teller (BET) specific surface area of 6.9 m^2^/g (WC) and 6.6 m^2^/g (WC-Co). A mean particle size 56 and 62 nm was calculated from BET values (d_BET_) for WC and WC-Co, respectively. By dynamic light scattering a mean particle size of 145 ± 5 nm for both WC and WC-Co was estimated (calculated according to DIN ISO 13321 [69]). Similar ranges of particle size distribution and morphology were measured for both types of particles. Particle size was shown to be stable in cell culture media supplemented with FBS (see also [21]). One week after the preparation of the suspension about 6% of tungsten from WC and 15% of tungsten and 76% of cobalt from WC-Co were found to be dissolved [21].

Exposure of cells to nanoparticles was performed using stock suspensions of 300 μg/ml WC (in water) and 330 μg/ml WC-Co (consisting of 300 μg/ml WC and 30 μg/ml cobalt, in 0.03% sodium polyphosphate, see Bastian et al. [21]). Particle suspensions were sterilised by autoclaving and treated for 10 min with ultrasound (Merck Eurolab, Darmstadt, Germany) to disperse aggregates before exposure of cells. Previous investigations did not reveal any changes in particle characteristics by autoclaving and re-dispersing [21].

A cobalt chloride (Fluka/Sigma-Aldrich, Seelze, Germany) stock solution of 10 mM was prepared in distilled water, sterilised by autoclaving and diluted with cell culture grade water (PAA Laboratories, Pasching, Austria). All suspensions and solutions were kept at 4°C.

### HaCaT cell culture and exposure of cells

The permanent human keratinocyte cell line, HaCaT (purchased from CLS - Cell Lines Service, Eppelheim, Germany) [70], was maintained in RPMI medium ('Roswell Park Memorial Institute' medium; Biochrom, Karlsruhe, Germany) supplemented with 5% (v/v) FBS and 1% (v/v) penicillin/streptomycin. Cells were cultured in monolayers at 37°C in a humidified, 5% (v/v) CO_2_-atmosphere and sub-cultured twice a week in 75 cm^2 ^flasks (Techno Plastic Products AG, Trasadingen, Switzerland); passages 30 to 40 were used for experiments. For sub-culturing, cells were washed three times with Versene (Invitrogen/Gibco, Berlin, Germany) and detached by trypsin (0.25% (v/v) in phosphate-buffered saline (Biowest, Renningen, Germany).

Cells were counted using a haemocytometer and seeded at densities of 2 × 10^5 ^cells/ml for 3 d of exposure or 5 × 10^5 ^cells/ml for 3 h of exposure, respectively in a final volume of 10 ml per 75 cm^2 ^flasks. In order to synchronise proliferation prior to exposure with nanoparticles, cells were allowed to grow for 24 h in RPMI with 5% FBS and subsequently for 24 h in RPMI without FBS for synchronisation [71]. Subsequently, cells were exposed to 30 μg/ml WC, 33 μg/ml WC-Co (cobalt content was 3 μg/ml), or 3 μg/ml cobalt chloride by mixing RPMI containing 5% FBS with 10 fold concentrated stock solutions. Exposure was performed in the dark with 5 independent replicates (performed at different days using different cell passage numbers).

Controls were performed with the water used for the preparation of particle suspensions. The WC-Co suspension also contained polyphosphate (0.003% v/v). However, polyphosphate was not included in controls since the final polyphosphate concentration did not exceed the normal sodium phosphate concentration in cell culture media. Furthermore, no evidences for any effect of low polyphosphate concentrations on cell vitality and function was observed in a previous study [21]. As also shown previously [21], nanoparticles did not aggregate during the exposure period if exposure was performed in FBS supplemented cell culture medium.

### RNA extraction

Total RNA was extracted from 75 cm^2 ^cell culture flasks with 1 ml Trizol reagent (Invitrogen, Karlsruhe, Germany) according to the manufacturer's instructions. RNA samples were additionally purified using the RNeasy Kit (Qiagen, Hilden, Germany), RNA qualities and quantities were determined with the Experion detection system (Biorad, Munich, Germany).

### Microarray experiments

The effect of the different treatments on transcription profiles of HaCaT cells was compared by microarray analysis (whole genome human 44K array, Agilent Technologies, Böblingen, Germany). Therefore, microarray hybridisations were performed for each treatment (control, WC 30 μg/ml, WC-Co 33 μg/ml, CoCl_2 _3 μg/ml; 3 h and 3 d exposure each) with 5 independent biological replicates. All hybridisations were performed against a common reference RNA [72] consisting of a mixture of equal amounts of RNA from all treatments. Synthesis of cDNA, cRNA and cRNA-labeling was performed with the Agilent Low RNA Input Linear Amplification Kit according to the manufacturer's instructions. cRNA was labelled with Cy3 (controls and treatments) and Cy5 (common reference). Cy3 and Cy5 labelled cRNA were combined and hybridised to the microarray slides in the DNA Microarrays Hybridisation Oven (Agilent Technologies). Slides were scanned with the Agilent DNA Microarray Scanner (Agilent Technologies). Hybridisation and scanning were performed according to standard protocols of the manufacturer.

### Microarray data analysis

Dye-normalised fluorescent intensities of individual microarray spots were extracted using the Agilent Feature Extraction software 9.5. Data were further normalised by dividing the Cy3/Cy5 ratio of each treatment by the mean Cy3/Cy5 ratio of the controls. Data were then analysed using the TMEV software version 4.3 (http://www.tm4.org/) [73]. Genes with significantly altered expression patterns were identified by a modified t-statistic (SAM = significance analysis of microarrays) [74]. Multiple comparison of the complete data set was performed using the lowest possible false discovery rate that allows identification of significantly differentially expressed gene (FDR < 0.03%). Further descriptive analysis by hierarchical clustering (TMEV 4.3) and principal component analysis (PCA, JMP 8.0, SAS institute; http://www.jmp.com) was restricted to the statistically significant genes. Fold changes (FC) of expression levels were calculated using the mean values of each treatment and the mean of the respective controls. A complete list of FC-values of all significantly differentially expressed genes is included in the supplementary information section of this paper (Additional file 1). The microarray data have been submitted to the Gene Expression Omnibus (GEO) database (series no. GSE16727, http://www.ncbi.nlm.nih.gov/geo/query/acc.cgi?acc=GSE16727).

### Gene set enrichment and pathway analysis

In order to identify biological pathways and functions associated with the changes in gene expression patterns, transcription profiles were analysed by Gene Set Enrichment Analysis (GSEA) [34,35](http://www.broad.mit.edu/gsea/). GSEA is based on ranking of the genes according to their statistical significance and comparison of the patterns to sets of predefined genes. These predefined gene sets are provided by the Molecular Signatures Database (MSigDB) and include five different types of databases (C1 to C5). For our analyses we used the databases C2 (gene sets collected from various sources such as online pathway databases, publications in PubMed including microarray studies, and knowledge of domain experts), C3 (transcription factor targets, i.e. genes that share a transcription factor binding site defined in the TRANSFAC database version 7.4, http://www.gene-regulation.com/) and C5 (gene sets of the Gene Ontology (GO) database, http://www.geneontology.org). Further details are explained on the MSigDB homepage http://www.broad.mit.edu/gsea/msigdb/index.jsp. Since GSEA does not allow the analysis of multiple datasets, analysis was performed pair wise comparing each treatment with the control.

Furthermore, pathway analysis was performed by means of the Database for Annotation, Visualisation and Integrated Discovery (DAVID) [36] (http://david.abcc.ncifcrf.gov/)using the list of differentially expressed genes identified by SAM (see above).

### RT-PCR

cDNA was synthesised from RNA using the RevAid™ First Strand cDNA Synthesis Kit (MBI Fermentas, St. Leon-Rot, Germany) according to the manufacturer's instructions. Primers were designed using the computer program Primer3 [75] or Beacon Designer 7 (Premier Biosoft, Palo Alto, USA; (http://www.PremierBiosoft.com) and purchased from Invitrogen. Primer sequences are listed in Table 4.

**Table 4:** Sequences of primers used for the validation of microarray data by RT-PCR

Gene Name	GenBank Accession	Forward Primer Sequence	Reverse Primer Sequence
RPL41	NM_021104	AAGATGAGGCAGAGGTCCAA	TCCAGAATGTCACAGGTCCA
LOXL2	NM_002318	AGCTTCTGCTTGGAGGACACA	TGAAGGAACCACCTATGTGGCA
ANGPTL4	NM_139314	GTCCTCGCACCTGGAACCC	CTTCGGGCAGGCTTGGCCAC
PFKFB4	NM_004567	TCCCCACGGGAATTGACAC	GAGAGTTGGGCAGTTGGTCAT
BNIP3	NM_004052	ACACCACAAGATACCAACAGG	TCTTCATGACGCTCGTGTTCCTC
GAPDH	NM_002046	AGGCTGAGAACGGGAAGC	AGAGGGGGCAGAGATGATG
CA9	NM_001216	AACCAGACAGTGATGCTGAGT	TGGCATAATGAGCAGGACAGGA
MAL	NM_002371	AAACATTGCTGCCGTGGTGTTC	AGGTTAGACACAGCAAGCTCCCA
OLFM4	NM_006418	ATTGGGTGGCGCCATTGAATA	TGGTGTTCATAGTACGGGTGGCA
ID2	NM_002166	GACCCGATGAGCCTGCTATAC	AATAGTGGGATGCGAGTCCAG
DSG4	NM_177986	TGAAGATGAAGGTCGACCAGC	GGGTTGCACACATGGATCAGCAT
KRT1	NM_006121	AGAATGCCCTCAAGGATGCCA	TTCTCCGGTAAGGCTGGGACAAA
MMP1	NM_002421	AAGAGGCTGGGAAGCCATCAC	TCAGTGAGGACAAACTGAGCCA

Target genes and the reference gene RPL41 [76] were amplified from 1 μl of cDNA using 1 unit of Taq Polymerase (Promega, Mannheim, Germany), 50 mM TRIS-HCl (pH 9.0, Serva, Heidelberg, Germany), 1.5 mM MgCl_2 _(Sigma, Steinheim, Germany), 15 mM (NH_4_)_2_SO_4 _(Sigma), 0.1% (v/v) Triton-X 100 (Merck, Darmstadt, Germany), 0.2 mM dNTPs (MBI Fermentas) and 0.6 μM of each primer in a 25 μl reaction volume. The number of cycles was adjusted to obtain amplified DNA during the exponential phase of the reaction. Annealing was performed at 55°C. PCR-fragments were analysed by agarose gel electrophoresis (1.5% w/v agarose) and ethidium bromide staining (0.005% w/v). mRNA abundance was evaluated by either visual comparison of band intensity or densitometric analysis using the image analysis software ImageJ (Version 1.33u, available at http://rsb.info.nih.gov/ij/). Relative gene expression levels were calculated by normalisation of band intensities to the reference gene. These relative gene expression values were converted to percent of the average control values. Statistical differences were analysed after confirmation of normal distribution (Kolmogorov-Smirnov test) with one-way ANOVA followed by Dunnett's post test using GraphPad Prism 4.0 software (GraphPad Software, San Diego California USA, http://www.graphpad.com). Values of p < 0.05 were considered statistically significant.

## Abbreviations

BET: Brunauer-Emmett-Teller specific surface area; DLS: dynamic light scattering; FBS: foetal bovine serum; FC: fold change; FDR: false discovery rate; GO: gene ontology; GSEA: gene set enrichment analyses; HCA: hierarchical cluster analyses; IARC: International Agency for Research on Cancer; LOEC: lowest observed effect level; MSigDB: Molecular Signature Database; NOEC: no observed effect concentration; PCA: principal component analyses; PI3Ks: phosphoinositol-3-kinases; ROS: reactive oxygen species; RPMI: Roswell Park Memorial Institute medium; RT-PCR: reverse transcriptase polymerase chain reaction; SAM: significance analyses of microarrays; TFT: transcription factor targets; v: volume; w: weight; WC: tungsten carbide; WC-Co: tungsten carbide cobalt; xPCS: mean particle size

## Authors' contributions

WB carried out the experiments, the computational analyses and manuscript preparation. DK assisted with the cell culture and exposures. KS and SS conceived and supervised the study, participated in the design of the study and in manuscript preparation. SS helped with the data analyses. All authors have read and approved the manuscript.

## Supplementary Material

Additional file 1 Table contains all significantly altered expressed genes and fold changes for all treatments Click here for file

Additional file 2 Heat map of differentially expressed genes and hierarchical clustering of all replicates Click here for file

Additional file 3 Table contains all gene sets and pathways identified as enriched by GSEA and DAVID Click here for file
