# Supplementary material for: Tungsten carbide cobalt nanoparticles exert hypoxia-like effects on the gene expression level in human keratinocytes
Source: BMC Genomics. 2010 Jan 27;11:65. doi: 10.1186/1471-2164-11-65 (PMC2824725; doi:10.1186/1471-2164-11-65)

AddFile2_HeatMap.pdf

Hierarchical clustering of all replicates using the differentially expressed genes. Colours indicate induction (red) and repression (green) of genes compared to mean control intensities. Differentially expressed genes were identified by SAM.

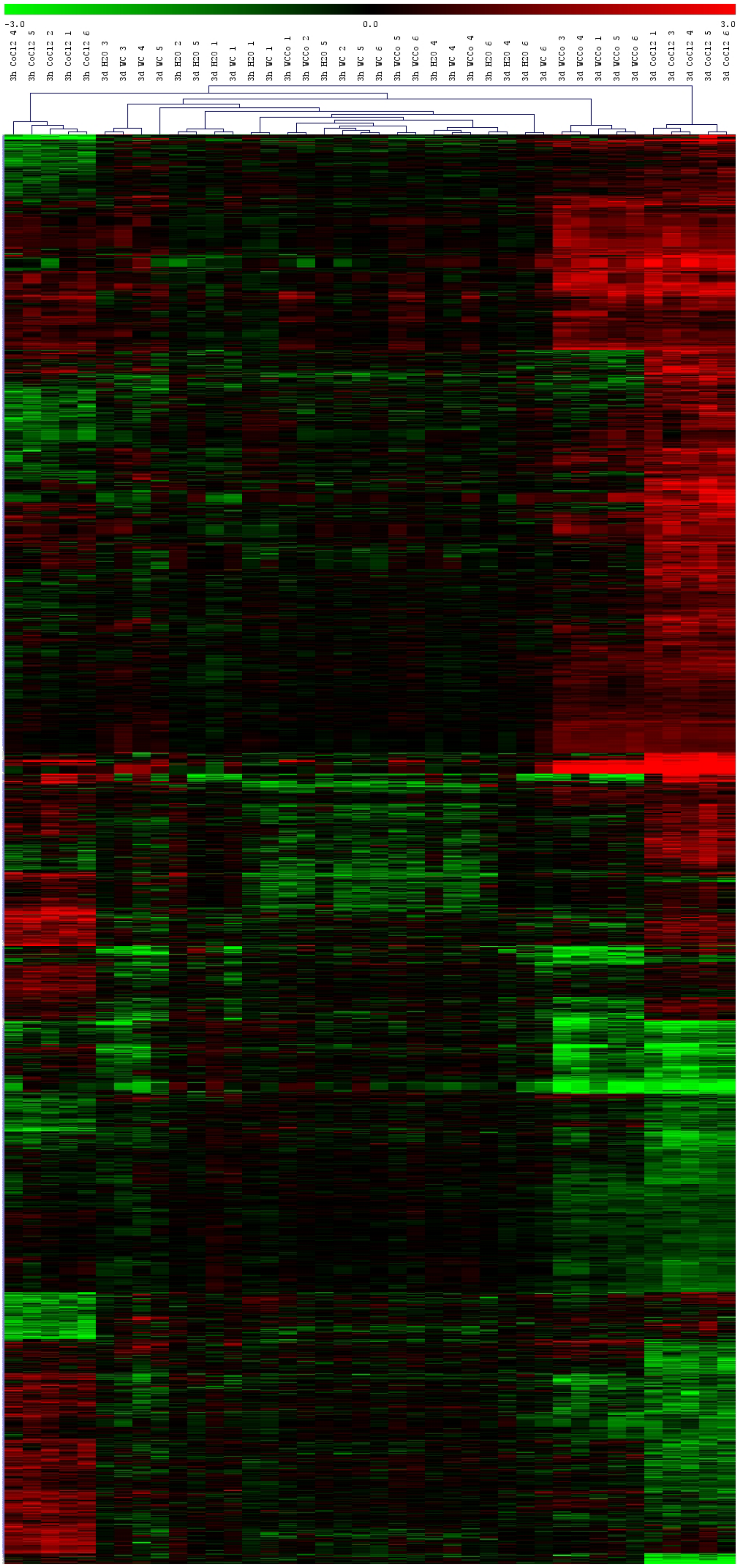

Supplement: Additional file 2 — Heat map of differentially expressed genes and hierarchical clustering of all replicates [file 1471-2164-11-65-S2.PDF]
